# Short-term outcomes of adding supervised joint mobilization to ultrasound-guided suprascapular nerve block plus home exercise for frozen shoulder: a single-center retrospective cohort study

**DOI:** 10.3389/fmed.2026.1943410

**Published:** 2026-09-17

**Authors:** Xi Chen, Chao Tian, Yu Zang, Xiaodong Zhang, Kangxiang Guo

**Affiliations:** 1 School of Medicine, Tongji University, Shanghai, China; 2 Department of Anesthesiology, Jing'an District Central Hospital, Shanghai, China; 3 Department of Rehabilitation Medicine, Zhangzhou Zhengxing Hospital, Zhangzhou, Fujian, China; 4 Department of Anesthesiology, School of Medicine, Yangpu Hospital, Tongji University, Shanghai, China; 5 Department of Rehabilitation Medicine, Hengdian Wenrong Hospital, Dongyang, Zhejiang, China

**Keywords:** frozen shoulder, home exercise, joint mobilization, retrospective cohort, short-term outcomes, suprascapular nerve block

## Abstract

**Objective:**

To compare 4-week short-term outcomes associated with adding supervised joint mobilization to ultrasound-guided suprascapular nerve block (SSNB) plus home exercise in patients with frozen shoulder and to explore factors associated with poor treatment response.

**Methods:**

This single-center retrospective cohort study included 124 patients with frozen shoulder who received SSNB between January 2024 and December 2024. The basic-treatment group received SSNB plus home exercise (*n* = 58), and the combined-treatment group received the same basic treatment plus supervised joint mobilization (*n* = 66). VAS pain score, shoulder range of motion, Constant-Murley Score (CMS), Shoulder Pain and Disability Index (SPADI), clinical response, and adverse events were evaluated at baseline and/or at 4 weeks. Continuous 4-week outcomes were analyzed by ANCOVA with adjustment for the corresponding baseline value and age; poor response was additionally analyzed by logistic regression adjusted for age and baseline VAS.

**Results:**

After adjustment for the corresponding baseline value and age, compared with the basic-treatment group, the combined-treatment group had a lower VAS score [adjusted mean difference (AMD), −0.78; 95% CI, −1.37 to −0.19; *P* = 0.010], greater forward flexion, abduction, and external rotation (AMD, 4.78°, 4.03°, and 3.38°, respectively; all *P* < 0.05), and a lower SPADI total score (AMD, −6.80; 95% CI, −11.08 to −2.53; *P* = 0.002). The adjusted between-group difference in CMS total score was not statistically significant (AMD, 3.42; 95% CI, −0.50 to 7.35; *P* = 0.087). The overall response rate was 89.39% in the combined-treatment group and 70.69% in the basic-treatment group; after adjustment for age and baseline VAS, combined treatment was associated with lower odds of poor response (adjusted OR, 0.319; 95% CI, 0.117–0.868; *P* = 0.025). Adverse-event rates did not differ significantly between groups (*P* = 0.147).

**Conclusion:**

In this single-center retrospective cohort, adding supervised joint mobilization to SSNB plus home exercise was associated with better 4-week pain, range-of-motion, and SPADI outcomes after adjustment for age and corresponding baseline values; the adjusted CMS difference was not statistically significant. These findings represent short-term associations and do not establish causality or long-term benefit.

## 1 Introduction

Frozen shoulder, also known as adhesive capsulitis, is a common shoulder disorder characterized by pain and progressive restriction of active and passive range of motion; the condition can persist for months to years and substantially impairs patients’ daily activities and work capacity (1). Epidemiological studies indicate that frozen shoulder affects approximately 2%–5% of the general population, occurs most often between 40 and 65 years of age, is slightly more common in women, and preferentially affects the non-dominant side (2). Diabetes mellitus and thyroid disease are well-recognized metabolic risk factors: the risk of frozen shoulder is 3- to 5-fold higher in patients with diabetes than in those without, and diabetic patients also tend to have a poorer treatment response and prognosis (3, 4). Conservative treatment remains the mainstay of management and includes oral analgesics, physical therapy, intra-articular or suprascapular nerve block, capsular distension, and surgical release (5).

Suprascapular nerve block (SSNB) can reduce shoulder pain by interrupting major sensory input and thereby facilitate active exercise and rehabilitation (6–8). Joint mobilization applies graded gliding and stretching to restricted capsular and periarticular tissues and may further improve range of motion and function (9–11). Previous studies suggest that adjunctive treatment added to injection or neural interventions may improve pain and range of motion, although the intervention combinations, follow-up periods, and study designs vary substantially (12). In the present study, both groups received SSNB and home exercise; the actual comparison was whether therapist-delivered, structured Maitland grade III-IV joint mobilization was added to this common basic treatment, rather than movement versus no movement or SSNB versus mobilization alone. We therefore retrospectively compared 4-week short-term outcomes between the two groups and explored factors associated with poor treatment response to inform future prospective research and clinical decision-making.

## 2 Materials and methods

### 2.1 General information

This single-center retrospective cohort study was approved by the Medical Ethics Committee of Shanghai Jing'an District Central Hospital (ethical approval number 202529) and was conducted in accordance with the Declaration of Helsinki. Clinical data were retrieved from the hospital information system and outpatient records for patients with frozen shoulder who received ultrasound-guided SSNB at the pain management and orthopedics outpatient clinic of Shanghai Jing'an District Central Hospital between January 2024 and December 2024.

Inclusion criteria were: (1) a clinical diagnosis of frozen shoulder (adhesive capsulitis) (1), based on history and physical examination showing restriction of both active and passive range of motion; (2) age 35–70 years; (3) treatment with ultrasound-guided SSNB and completion of the 4-week outcome assessment; and (4) complete medical and follow-up records.

Exclusion criteria: (1) secondary frozen shoulder attributable to rotator cuff tear, fracture-dislocation, glenohumeral osteoarthritis, or prior shoulder surgery; (2) concurrent shoulder infection, tumor, or other organic disease; (3) severe cardiopulmonary insufficiency, coagulopathy, or other contraindication to nerve block; (4) prior invasive intervention, such as intra-articular injection or extracorporeal shock-wave therapy, before the index treatment; (5) incomplete data or loss to follow-up.

A total of 163 patients were screened, and 124 remained after application of the exclusion criteria. All eligible patients with complete records during the study period were included; no prospective sample-size calculation based on an anticipated effect size was performed. Patients were grouped according to the treatment actually received: the basic-treatment group received SSNB plus home exercise (*n* = 58), whereas the combined-treatment group received SSNB plus home exercise plus supervised joint mobilization (*n* = 66). The patient-selection flow chart is shown in Figure 1.

**Figure 1:**
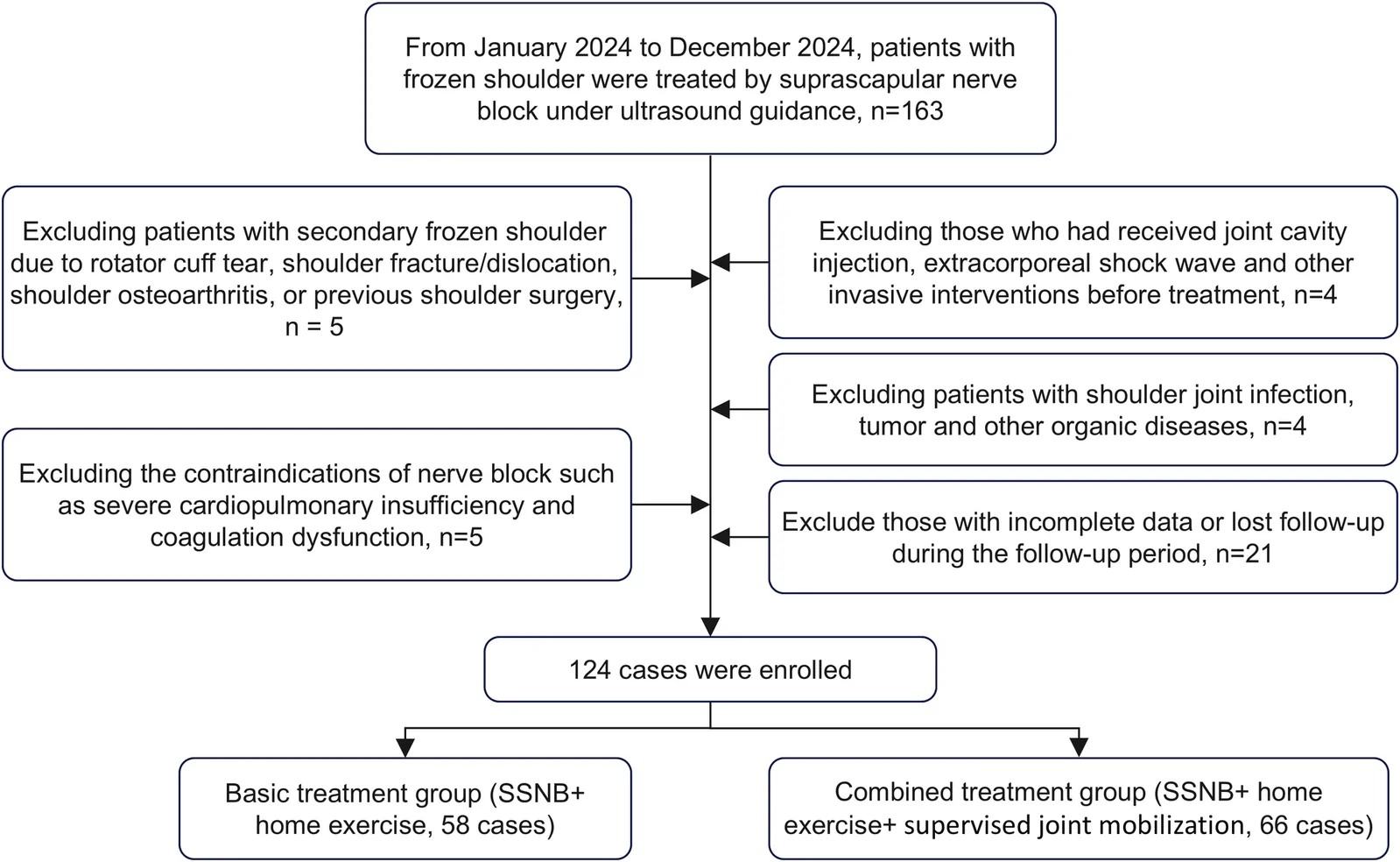
Flow chart of patient screening and group allocation. SSNB, suprascapular nerve block.

This study was not randomized. Treatment selection arose from routine shared decision-making between patients and clinicians and may have been influenced by baseline clinical status, patient preference, access to rehabilitation, ability to attend scheduled sessions, and clinician judgment. The available records could not fully reconstruct the reason for treatment choice for every patient; selection bias and confounding by indication are therefore possible.

### 2.2 Treatment protocol

Both groups received the same ultrasound-guided SSNB protocol and home exercise program. For SSNB, patients were seated with the affected upper limb adducted and internally rotated. A high-frequency linear-array ultrasound probe was used to identify the suprascapular nerve and accompanying vessels in the supraspinous fossa. Using an in-plane approach, the needle was advanced to the perineural region, and after negative aspiration for blood, 5 mL of 0.375% ropivacaine mixed with 1 mL of compound betamethasone was injected. SSNB was performed once weekly for 4 consecutive weeks. During treatment, patients in both groups were instructed to perform home exercises, including Codman pendulum and wall-climbing exercises.

The combined-treatment group additionally received supervised joint mobilization. After the block had taken effect and pain had decreased, a rehabilitation therapist trained in the Maitland concept applied grade III-IV mobilization in the directions of restricted forward flexion, abduction, external rotation, and internal rotation (3–5 sets per direction, 30 s per set), together with posterior and inferior capsular glides. Each session lasted 20–30 min and was performed three times weekly for 4 consecutive weeks. The basic-treatment group did not receive therapist-delivered structured joint mobilization.

Operators and quality control: Ultrasound-guided SSNB was performed by five pain-medicine/orthopedic physicians, all at attending-physician level or above, with 3–15 years of experience in ultrasound-guided interventional procedures. Joint mobilization was delivered by three rehabilitation therapists who had received standardized Maitland training and had 2–9 years of relevant clinical experience. To reduce operator variability, standardized protocols were used throughout the study for patient positioning, ultrasound localization, needle trajectory, drug type and dose, treatment frequency, mobilization timing and grade, treatment direction, set duration, frequency, and course. Treatment records were cross-checked against the medical record system, which retained operator information. Because the number of operators was small, case volumes were uneven, and only 24 poor-response events occurred, adding operator as a covariate to the main multivariable model could further increase overfitting; operator was therefore not included in the primary models. Standardization may reduce but cannot eliminate operator-related variation.

### 2.3 Outcome measures

(i) Pain intensity was assessed using the Visual Analog Scale (VAS), scored from 0 to 10, with higher scores indicating greater pain. (ii) Active forward flexion, abduction, and external rotation of the affected shoulder were measured with a goniometer. (iii) Shoulder function was assessed using the Constant-Murley Score (CMS) (13), which comprises pain (0–15), activities of daily living (0–20), range of motion (0–40), and muscle strength (0–25), for a total score of 0–100; higher scores indicate better function. (iv) Shoulder pain and disability were assessed using the Shoulder Pain and Disability Index (SPADI) (14), which contains pain and disability domains and yields a total score from 0 to 100; higher scores indicate greater pain and disability. (v) Clinical response at 4 weeks was categorized exploratorily according to the percentage improvement in VAS score: VAS improvement rate = (baseline score - 4-week score)/baseline score×100%.

Marked improvement was defined as a VAS improvement rate >=70%, improvement as 30% to <70%, and no improvement (poor response) as <30%. A 30% reduction in pain can represent a moderately clinically important improvement (15); the 70% threshold was an operational cutoff used in this study to distinguish “marked improvement” and is not a universally validated multidimensional response criterion for frozen shoulder. This categorical response was therefore treated as an auxiliary outcome, while CMS, SPADI, and range of motion were used to capture functional outcomes. Overall response rate = (marked improvement + improvement)/total number of patients×100%. (vi) Adverse events recorded during treatment included local hematoma, dizziness/vasovagal reaction, infection, and transient neurologic irritation symptoms. Outcomes (i)-(iv) were assessed at baseline and at 4 weeks; outcomes (v) and (vi) were summarized at 4 weeks. Because the data were derived from routine outpatient records, blinding of outcome assessors to treatment group was not consistently documented and could not be confirmed.

### 2.4 Statistical analysis

Data were entered in Excel and analyzed using SPSS version 26.0. Continuous variables are presented as mean ± standard deviation; unadjusted between-group comparisons used independent-samples t tests, and within-group baseline-to-4-week comparisons used paired-samples t tests. Categorical variables are presented as *n* (%) and were compared using the chi-square test. To address the baseline age imbalance and differences in baseline outcome levels, separate ANCOVA models were fitted for the 4-week VAS score, forward flexion, abduction, external rotation, CMS total score, and SPADI total score, with treatment group as the fixed factor and the corresponding baseline outcome value and age as covariates. Homogeneity of regression slopes was examined using the treatment-group × baseline-value interaction. Adjusted estimated marginal means, adjusted mean differences (combined-treatment group minus basic-treatment group), and 95% CIs were reported. Sensitivity analyses additionally adjusted for disease duration, diabetes, hypertension, and affected side; covariates were selected on clinical grounds rather than solely by baseline *P* values. Home-exercise adherence during treatment was not included in the primary adjusted outcome models because it could lie on the treatment pathway. ANCOVA residual distributions and homoscedasticity were examined, and HC3 heteroscedasticity-robust standard errors were used in sensitivity analyses. For the adjusted analysis of overall response, poor response (yes=1, no = 0) was modeled using multivariable logistic regression including treatment group, age, and baseline VAS; adjusted odds ratios (aORs) and 95% CIs were reported. A separate exploratory analysis of factors associated with poor response first used univariate analyses and then included treatment group, diabetes, hypertension, home-exercise adherence, age, and disease duration >7 months in a multivariable unconditional logistic model. Because only 24 poor-response events occurred, this latter model was considered hypothesis-generating rather than confirmatory or causal. All tests were two-sided with *P* < 0.05 considered statistically significant. No multiplicity adjustment was applied across the multiple outcomes; corresponding *P* values should therefore be interpreted as exploratory.

## 3 Results

### 3.1 Baseline characteristics

Sex, BMI, disease duration, affected side, diabetes, hypertension, thyroid disease, occupation, education level, smoking history, and adherence to home exercise did not differ significantly between groups (all *P* > 0.05). The combined-treatment group was younger than the basic-treatment group (*P* = 0.005). The between-group *P* values for affected side, diabetes, and hypertension were 0.064, 0.059, and 0.061, respectively; although these did not cross the 0.05 threshold, they were considered potentially relevant confounders. Accordingly, the primary ANCOVA models adjusted for the corresponding baseline outcome and age, and expanded sensitivity models additionally included disease duration, diabetes, hypertension, and affected side (Table 1).

**Table 1:** Baseline characteristics of the two groups [mean ± SD/n (%)].

Item	Category	Basic-treatment group (*n* = 58)	Combined-treatment group (*n* = 66)	t/*χ*^2^	P
Age (years)		56.02 ± 6.21	52.73 ± 6.64	2.851	0.005
BMI (kg/m^2^)		24.72 ± 2.52	25.16 ± 2.52	0.975	0.332
Disease duration (months)		7.43 ± 2.78	7.23 ± 3.20	0.366	0.715
Sex	Male	26 (44.83)	29 (43.94)	0.010	0.921
	Female	32 (55.17)	37 (56.06)		
Affected side	Dominant	41 (70.69)	36 (54.55)	3.419	0.064
	Non-dominant	17 (29.31)	30 (45.45)		
Comorbid diabetes	Yes	18 (31.03)	11 (16.67)	3.557	0.059
	No	40 (68.97)	55 (83.33)		
Comorbid hypertension	Yes	10 (17.24)	21 (31.82)	3.498	0.061
	No	48 (82.76)	45 (68.18)		
Comorbid thyroid disease	Yes	6 (10.34)	7 (10.61)	0.002	0.962
	No	52 (89.66)	59 (89.39)		
Occupation (manual labor)	Yes	17 (29.31)	29 (43.94)	2.831	0.092
	No	41 (70.69)	37 (56.06)		
Education level	Primary school or below	14 (24.14)	25 (37.88)	3.596	0.166
	Secondary school	28 (48.28)	30 (45.45)		
	College or above	16 (27.59)	11 (16.67)		
Smoking history	Yes	11 (18.97)	20 (30.30)	2.116	0.146
	No	47 (81.03)	46 (69.70)		
Good adherence to home exercise	Yes	26 (44.83)	38 (57.58)	2.009	0.156
	No	32 (55.17)	28 (42.42)		

Values are mean ± SD for continuous variables and *n* (%) for categorical variables. BMI, body mass index.

### 3.2 VAS pain scores before and after treatment

Before treatment, VAS scores did not differ significantly between groups (*P* > 0.05). At 4 weeks, VAS scores had decreased in both groups relative to baseline, and the combined-treatment group had a lower unadjusted VAS score than the basic-treatment group (*P* < 0.001) (Figure 2).

**Figure 2:**
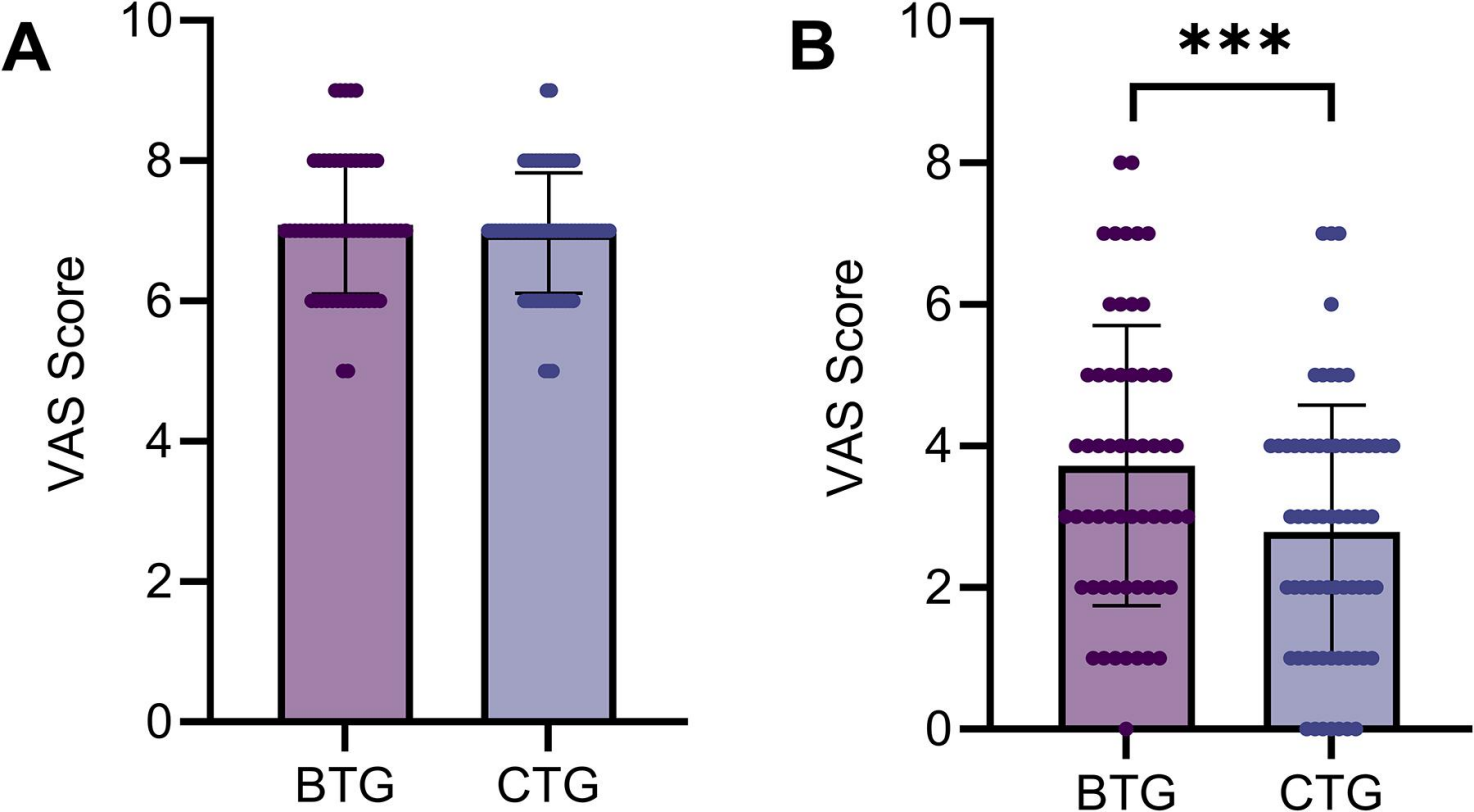
VAS pain scores before and after treatment. At baseline **(A)**, VAS scores did not differ significantly between groups (*P* > 0.05); at 4 weeks **(B)**, the combined-treatment group had a lower unadjusted VAS score than the basic-treatment group (*P* < 0.001). BTG, basic-treatment group (SSNB + home exercise); CTG, combined-treatment group (SSNB + home exercise + supervised joint mobilization); ***P < 0.001.

### 3.3 Shoulder range of motion before and after treatment

At baseline, forward flexion, abduction, and external rotation did not differ significantly between groups (all *P* > 0.05). At 4 weeks, all three measures had improved significantly within both groups (all *P* < 0.05). In unadjusted between-group comparisons, forward flexion and external rotation were greater in the combined-treatment group than in the basic-treatment group (both *P* < 0.05), whereas the difference in abduction did not reach statistical significance (*P* = 0.051) (Figure 3).

**Figure 3:**
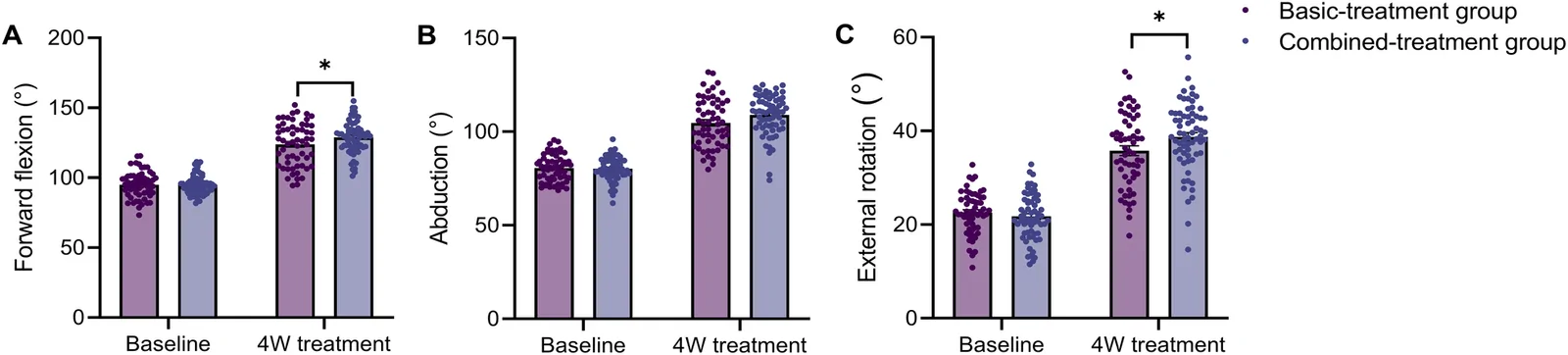
Shoulder range of motion before and after treatment. At baseline, forward flexion **(A)**, abduction **(B)**, and external rotation **(C)** did not differ significantly between groups (*P* > 0.05). At 4 weeks, unadjusted forward flexion and external rotation were greater in the combined-treatment group (*P* < 0.05), whereas the between-group difference in abduction was not statistically significant (*P* = 0.051). BTG, basic-treatment group; CTG, combined-treatment group; BT, baseline; AT 4W, after 4 weeks; *P < 0.05.

### 3.4 Constant-Murley scores before and after treatment

At baseline, total CMS and all domain scores did not differ significantly between groups (all *P* > 0.05). At 4 weeks, total CMS and all domain scores had improved significantly within both groups (all *P* < 0.001), and the combined-treatment group had higher unadjusted total and domain scores than the basic-treatment group (all *P* < 0.05) (Table 2).

**Table 2:** Constant-Murley score (CMS) before and after treatment (points, mean ± SD).

Domain	Basic-treatment Baseline	Basic-treatment 4 weeks	Combined-treatment Baseline	Combined-treatment 4 weeks	*P* (baseline)	*P* (4 wk)
Pain (0–15)	5.29 ± 0.77	9.03 ± 2.19	5.47 ± 0.73	9.91 ± 1.69	0.195	0.015
Activities of daily living (0–20)	7.05 ± 1.07	12.17 ± 2.90	7.33 ± 1.03	13.23 ± 2.22	0.138	0.026
Range of motion (0–40)	14.16 ± 2.08	24.28 ± 5.69	14.86 ± 2.11	26.50 ± 4.29	0.062	0.017
Muscle strength (0–25)	8.78 ± 1.28	15.07 ± 3.52	9.17 ± 1.38	16.56 ± 2.64	0.105	0.010
Total score (0–100)	35.28 ± 5.04	60.55 ± 14.25	36.83 ± 5.08	66.20 ± 10.76	0.090	0.015

Within-group comparisons (baseline vs. 4 weeks) were significant for all domains in both groups (all *P* < 0.001, not shown in the table). P (baseline) and P (4 wk) denote unadjusted between-group comparisons at baseline and at 4 weeks, respectively.

### 3.5 SPADI scores before and after treatment

At baseline, SPADI pain, disability, and total scores did not differ significantly between groups (all *P* > 0.05). At 4 weeks, all SPADI domain and total scores had decreased significantly within both groups (all *P* < 0.001), and the combined-treatment group had lower unadjusted pain, disability, and total scores than the basic-treatment group (all *P* < 0.05) (Table 3).

**Table 3:** Shoulder pain and disability Index (SPADI) scores before and after treatment (points, mean ± SD).

Domain	Basic-treatment Baseline	Basic-treatment 4 weeks	Combined-treatment Baseline	Combined-treatment 4 weeks	P (baseline)	P (4 wk)
Pain	69.62 ± 6.88	33.29 ± 15.74	68.82 ± 8.49	25.41 ± 15.78	0.562	0.006
Disability	65.58 ± 7.96	31.51 ± 13.47	64.91 ± 6.54	24.00 ± 11.13	0.613	0.001
Total score	67.60 ± 5.34	32.40 ± 13.49	66.88 ± 5.30	24.71 ± 11.56	0.451	<0.001

Within-group comparisons (baseline vs. 4 weeks) were significant for all domains in both groups (all *P* < 0.001, not shown in the table).

### 3.6 Adjusted analysis of primary continuous outcomes

Treatment-group × baseline-value interaction terms were not statistically significant for any outcome (*P* = 0.458–0.992), supporting the homogeneity-of-regression-slopes assumption. After adjustment for the corresponding baseline value and age, the combined-treatment group had lower 4-week VAS and SPADI total scores and greater forward flexion, abduction, and external rotation than the basic-treatment group (all *P* < 0.05). The adjusted CMS total score was higher in the combined-treatment group, but the between-group difference was not statistically significant (AMD=3.42; 95% CI, −0.50 to 7.35; *P* = 0.087) (Table 4). Some model residuals deviated from normality; however, HC3 heteroscedasticity-robust standard errors did not change the statistical inferences. Further adjustment for disease duration, diabetes, hypertension, and affected side produced the same effect direction and statistical inference for VAS, forward flexion, abduction, external rotation, and SPADI; the CMS total-score difference remained nonsignificant (*P* = 0.141), supporting the overall stability of the main findings.

**Table 4:** Adjusted comparison of primary continuous outcomes at 4 weeks.

Outcome	Basic-treatment adjusted mean (95% CI)	Combined-treatment adjusted mean (95% CI)	AMD	95% CI	P
VAS score	3.64 (3.22–4.06)	2.86 (2.47–3.25)	−0.78	−1.37 to −0.19	0.010
Forward flexion (°)	124.07 (121.24–126.90)	128.84 (126.20–131.49)	4.78	0.84 to 8.71	0.018
Abduction (°)	104.84 (102.34–107.34)	108.87 (106.53–111.20)	4.03	0.55 to 7.51	0.024
External rotation (°)	35.55 (33.91–37.18)	38.92 (37.40–40.45)	3.38	1.09 to 5.66	0.004
CMS total score	61.73 (58.92–64.55)	65.16 (62.53–67.79)	3.42	−0.50 to 7.35	0.087
SPADI total score	31.93 (28.86–35.00)	25.12 (22.25–27.99)	−6.80	−11.08 to −2.53	0.002

Values are adjusted estimated marginal means (95% CI). Each model used the corresponding 4-week outcome as the dependent variable, treatment group as the fixed factor, and adjusted for the same outcome at baseline and age. Adjusted mean difference (AMD) = combined-treatment group minus basic-treatment group; negative values for VAS and SPADI and positive values for range of motion and CMS favor the combined-treatment group.

### 3.7 Clinical response and adverse events

The overall response rate was higher in the combined-treatment group than in the basic-treatment group (89.39% vs. 70.69%; chi-square=6.919, *P* = 0.009) (Table 5). With poor response as the dependent variable, after adjustment for age and baseline VAS, combined treatment was associated with lower odds of poor response (aOR=0.319; 95% CI, 0.117–0.868; *P* = 0.025). At least one adverse event occurred in 10/66 patients (15.15%) in the combined-treatment group and 4/58 (6.90%) in the basic-treatment group; the between-group difference was not statistically significant (chi-square=2.100, *P* = 0.147). This nonsignificant result does not establish safety equivalence or exclude a clinically important risk difference. The available aggregate records did not permit reliable stratified reporting of adverse-event severity, relatedness, or timing.

**Table 5:** Clinical response in the two groups [n (%)].

Group	n	Marked improvement	Improvement	No improvement	Overall response rate (%)
Basic-treatment group	58	15 (25.86)	26 (44.83)	17 (29.31)	70.69
Combined-treatment group	66	25 (37.88)	34 (51.52)	7 (10.61)	89.39

Overall response-rate comparison: chi-square=6.919, *P* = 0.009.

### 3.8 Univariate analysis of factors associated with poor response

Using no improvement at 4 weeks as the definition of poor response, 24 of 124 patients (19.35%) had poor response and 100 (80.65%) had satisfactory response. Univariate analysis showed associations of treatment modality, diabetes, hypertension, and home-exercise adherence with poor response (all *P* < 0.05). Disease duration and occupation approached but did not reach statistical significance (*P* = 0.063 and *P* = 0.066, respectively), whereas age, sex, BMI, affected side, thyroid disease, education level, and smoking history were not significantly associated with poor response (all *P* > 0.05) (Table 6).

**Table 6:** Univariate analysis of factors associated with poor response in patients with frozen shoulder.

Item	Category	Poor-response group (*n* = 24)	Satisfactory-response group (*n* = 100)	t/χ^2^	P
Treatment modality	Basic-treatment group	17 (70.83)	41 (41.00)	6.919	0.009
	Combined-treatment group	7 (29.17)	59 (59.00)		
Age (years)		55.75 ± 6.90	53.91 ± 6.54	1.184	0.245
BMI (kg/m^2^)		25.06 ± 2.83	24.93 ± 2.45	0.214	0.832
Disease duration (months)		8.27 ± 2.59	7.10 ± 3.06	1.915	0.063
Sex	Male	9 (37.50)	46 (46.00)	0.567	0.452
	Female	15 (62.50)	54 (54.00)		
Affected side	Dominant	17 (70.83)	60 (60.00)	0.965	0.326
	Non-dominant	7 (29.17)	40 (40.00)		
Comorbid diabetes	Yes	11 (45.83)	18 (18.00)	8.368	0.004
	No	13 (54.17)	82 (82.00)		
Comorbid hypertension	Yes	10 (41.67)	21 (21.00)	4.409	0.036
	No	14 (58.33)	79 (79.00)		
Comorbid thyroid disease	Yes	2 (8.33)	11 (11.00)	0.147	0.702
	No	22 (91.67)	89 (89.00)		
Occupation (manual labor)	Yes	5 (20.83)	41 (41.00)	3.373	0.066
	No	19 (79.17)	59 (59.00)		
Education level	Primary school or below	8 (33.33)	31 (31.00)	0.342	0.843
	Secondary school	10 (41.67)	48 (48.00)		
	College or above	6 (25.00)	21 (21.00)		
Smoking history	Yes	7 (29.17)	24 (24.00)	0.276	0.600
	No	17 (70.83)	76 (76.00)		
Good adherence to home exercise	Yes	8 (33.33)	56 (56.00)	3.982	0.046
	No	16 (66.67)	44 (44.00)		

### 3.9 Exploratory multivariable logistic regression of factors associated with poor response

Poor response (yes=1, no = 0) was used as the dependent variable. Treatment modality (combined treatment=1, basic treatment=0), diabetes, hypertension, home-exercise adherence, age, and disease duration >7 months were entered into an exploratory multivariable logistic regression model. Diabetes (OR = 3.763; 95% CI, 1.261–11.227) and hypertension (OR = 4.186; 95% CI, 1.363–12.856) were associated with higher odds of poor response, whereas combined treatment was associated with lower odds (OR = 0.311; 95% CI, 0.103–0.934; all *P* < 0.05). Disease duration, home-exercise adherence, and age were not statistically significant in this model. Because only 24 poor-response events were available for six variables, the confidence intervals were wide and the model may be affected by overfitting; these findings should therefore be regarded as hypothesis-generating associations (Table 7).

**Table 7:** Exploratory multivariable logistic regression analysis of factors associated with poor response.

Variable	β	SE	Wald χ^2^	P	OR	95% CI
Treatment modality (combined treatment=1)	−1.169	0.561	4.336	0.037	0.311	0.103–0.934
Comorbid diabetes (yes=1)	1.325	0.558	5.647	0.017	3.763	1.261–11.227
Disease duration >7 months (yes=1)	0.298	0.519	0.330	0.566	1.347	0.487–3.723
Comorbid hypertension (yes=1)	1.432	0.573	6.254	0.012	4.186	1.363–12.856
Good adherence to home exercise (yes=1)	−0.831	0.523	2.527	0.112	0.435	0.156–1.214
Age (years)	0.006	0.041	0.022	0.883	1.006	0.928–1.090

## 4 Discussion

In this single-center retrospective cohort, both groups received SSNB and home exercise, and the addition of supervised Maitland grade III-IV joint mobilization was associated with better short-term outcomes at 4 weeks. Because the combined-treatment group was younger, we used ANCOVA to adjust each continuous outcome for its baseline value and age. After adjustment, the combined-treatment group had lower VAS and SPADI total scores and greater forward flexion, abduction, and external rotation; these inferences were unchanged in expanded covariate sensitivity analyses. Notably, the unadjusted between-group difference in CMS total score was statistically significant, but the adjusted difference was smaller and no longer statistically significant (AMD=3.42; 95% CI, −0.50 to 7.35; *P* = 0.087), indicating that the unadjusted CMS result may have been partly influenced by age or baseline outcome levels. The adjusted response analysis similarly showed that combined treatment was associated with lower odds of poor response (aOR=0.319). These findings support a possible short-term incremental benefit of structured mobilization on top of common basic treatment; however, because there was no mobilization-only group and treatment was not randomized, covariate adjustment can reduce only measured confounding and cannot demonstrate a true synergistic effect or a definitive causal treatment effect. Previous studies suggest that SSNB combined with exercise or other adjunctive interventions may improve pain and range of motion (6, 7, 12, 16, 17), but differences in design and intervention mean that these studies provide only indirect support. Plausible mechanisms include SSNB-mediated reduction of pain and protective muscle spasm, which may improve tolerance of subsequent rehabilitation, and progressive gliding and stretching during joint mobilization, which may reduce capsular and periarticular restriction (8–11); these mechanisms were not directly measured in the present study. Adverse-event rates did not differ statistically between groups, but the limited sample size does not establish equivalent safety.

In the exploratory regression, diabetes and hypertension were associated with higher odds of poor response, whereas combined treatment was associated with lower odds. Diabetes-related collagen cross-linking and capsular fibrosis may adversely affect treatment response (2–5). Potential mechanisms related to hypertension were not measured in this study and should therefore be interpreted cautiously. Good home-exercise adherence was associated with treatment response in univariate analysis but was not statistically significant in the multivariable model, suggesting possible confounding among treatment choice, comorbidities, and exercise behavior. Importantly, we did not test treatment-by-diabetes or treatment-by-hypertension interactions and did not perform adequately powered stratified analyses. The present data therefore do not show that patients with diabetes or hypertension derive a greater relative benefit from combined treatment. Clinical decisions may reasonably consider comorbidities, functional limitation, rehabilitation access, and patient preference, but any recommendation to prioritize combined treatment in specific high-risk subgroups requires dedicated subgroup-effect studies.

This study has several limitations. First, it was a single-center, retrospective, nonrandomized study. Treatment choice may have been influenced by baseline disease severity, patient preference, rehabilitation access, scheduling, and clinician judgment, creating potential selection bias, confounding by indication, and unmeasured confounding. Age differed significantly at baseline, and affected side, diabetes, and hypertension showed possible imbalance. Although the primary continuous outcomes were adjusted for the corresponding baseline value and age and were examined in expanded sensitivity models, residual confounding remains possible. Second, outcomes were assessed at week 4, when the repeated block and mobilization programs ended. These measurements therefore reflect immediate/short-term treatment response; recent effects of local anesthetic, corticosteroid, and concurrent treatment may still have been present, and no post-treatment medium- or long-term follow-up, recurrence, or durability data were available. Third, assessor blinding was not consistently documented. Multiple physicians and therapists delivered the interventions; standardized protocols were used, but operator effects were not separately adjusted for and observer or operator-related variability cannot be excluded. Fourth, formal normality assessments for all raw continuous variables were not consistently preserved in the retrospective analysis record, and some ANCOVA residuals deviated from normality. HC3 robust-standard-error sensitivity analyses produced the same statistical inferences, but the absence of multiplicity adjustment across multiple outcomes increases the risk of chance-positive findings. Fifth, only 24 poor-response events were available for six variables in the exploratory logistic model (approximately four events per variable), creating a risk of overfitting, wide confidence intervals, and unstable estimates; the 7-month disease-duration cutoff was also an exploratory dichotomization. Sixth, sample size was determined by all eligible cases during the study period, without a prospective power calculation; power was therefore limited for adverse-event differences, interaction tests, and subgroup effects. Seventh, reliable group-level detail on adverse-event type, severity, relatedness, and timing was not available, and metabolic indices such as glycemic control and thyroid function were not included. These limitations mean that the results should be interpreted as short-term associative evidence and require confirmation in multicenter prospective studies with longer follow-up and prespecified analysis plans.

## 5 Conclusion

In this single-center retrospective cohort, compared with SSNB plus home exercise, adding supervised joint mobilization was associated with lower VAS and SPADI scores and greater shoulder forward flexion, abduction, and external rotation at 4 weeks; these differences remained statistically significant after adjustment for the corresponding baseline outcome and age. The adjusted between-group difference in CMS total score was not statistically significant. After adjustment for age and baseline VAS, combined treatment remained associated with lower odds of poor response. Adverse-event rates did not differ statistically between groups, but this finding does not establish safety equivalence. Because treatment was nonrandomized and residual confounding is possible, these results represent short-term associations and do not support definitive causal inference or preferential treatment recommendations for specific high-risk subgroups. Prospective studies are needed to evaluate long-term effectiveness and safety.

## Data Availability

The original contributions presented in the study are included in the article; further inquiries can be directed to the corresponding author.
